# Supplementary material for: Splice-Junction-Based Mapping of Alternative Isoforms in the Human Proteome
Source: Cell Rep. Author manuscript; Available in PMC 2020 Jan 15. (PMC6961840; doi:10.1016/j.celrep.2019.11.026)
Supplement: 2 [file NIHMS1546469-supplement-2.zip › DF1/PXD009737/TestisLysC/TestisLysC_23_MLLT1_LLRAGGDANK.pdf]

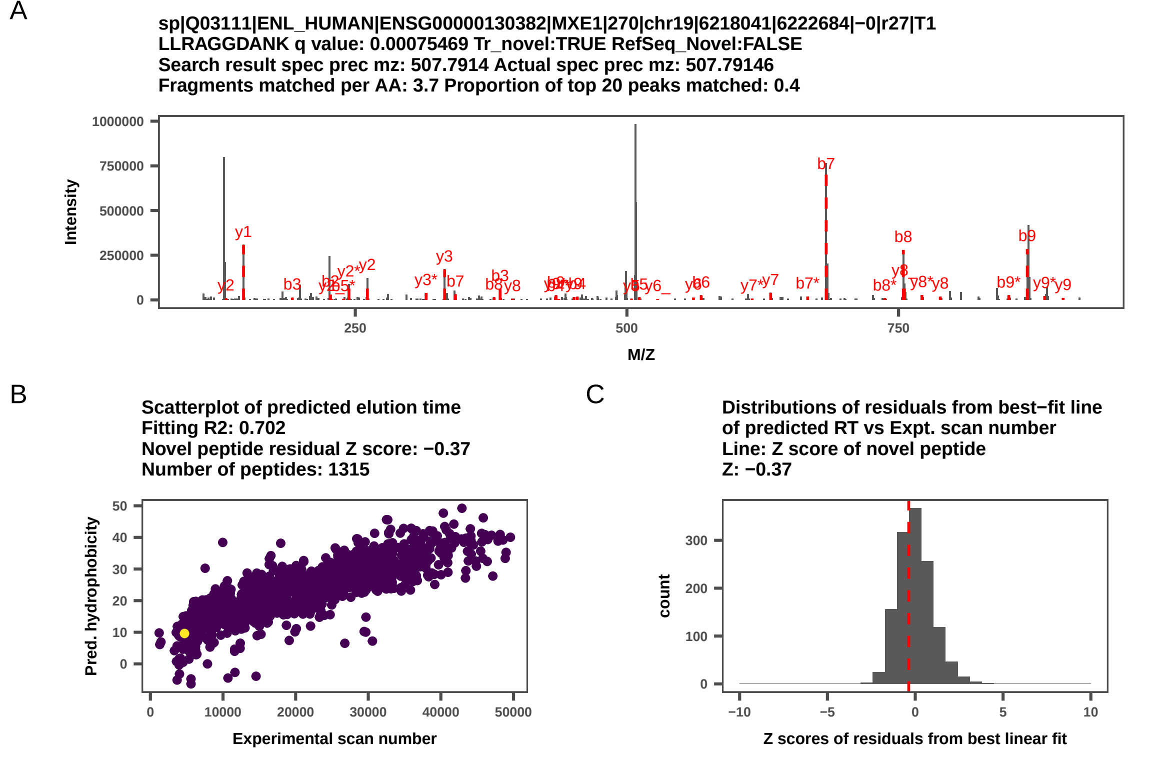
A
sp|Q03111|ENL_HUMAN|ENSG00000130382|MXE1|270|chr19|6218041|6222684|−0|r27|T1
LLRAGGDANK q value: 0.00075469 Tr_novel:TRUE RefSeq_Novel:FALSE
Search result spec prec mz: 507.7914 Actual spec prec mz: 507.79146
Fragments matched per AA: 3.7 Proportion of top 20 peaks matched: 0.4
Intensity
1000000
750000
500000
250000
0
250
500
750
M/Z
y1
y3
b7
b8
b9
B
Scatterplot of predicted elution time
Fitting R2: 0.702
Novel peptide residual Z score: −0.37
Number of peptides: 1315
Pred. hydrophobicity
50
40
30
20
10
0
0
10000
20000
30000
40000
50000
Experimental scan number
C
Distributions of residuals from best−fit line
of predicted RT vs Expt. scan number
Line: Z score of novel peptide
Z: −0.37
count
300
200
100
0
−10
−5
0
5
10
Z scores of residuals from best linear fit
